# Air quality and photochemical reactions: analysis of NO_x_ and NO_2_ concentrations in the urban area of Turin, Italy

**DOI:** 10.1007/s11869-022-01168-1

**Published:** 2022-02-11

**Authors:** Marco Ravina, Gianmarco Caramitti, Deborah Panepinto, Mariachiara Zanetti

**Affiliations:** Department of Environment, Land and Infrastructure Engineering, Turin Polytechnic, Corso Duca degli Abruzzi 24, 10129 Turin, Italy

**Keywords:** Air quality, Photochemical smog, Nitrogen oxides, Ozone, Atmospheric pollution

## Abstract

In this work, based on the existing studies on photochemical reactions in the lower atmosphere, an analysis of the historical series of NO_x_, NO_2_, and O_3_ concentrations measured in the period 2015–2019 by two monitoring stations located in the urban area of Turin, Italy, was elaborated. The objective was to investigate the concentration trends of the contaminants and evaluate possible simplified relationships based on the observed values. Concentration trends of these pollutants were compared in different time bands (diurnal or seasonal cycles), highlighting some differences in the dispersion of the validated data. Calculated [NO_2_]/[NO_x_] ratios were in agreement with the values observed in other urban areas worldwide. The influence of temperature on the [NO_2_]/[NO_x_] ratio was investigated. An increase of [NO_2_]/[NO_x_] concentration ratio was found with increasing temperature. Finally, a set of empirical relationships for the preliminary determination of NO_2_ concentration values as a function of the NO_x_ was elaborated and compared with existing formulations. Polynomial functions were adapted to the average concentration values returned by the division into classes of 10 μg/m^3^ of NO_x_. The choice of an empirical function to estimate the trend of NO_2_ concentrations is potentially useful for the preliminary data analysis, especially in case of data scarcity. The scatter plots showed differences between the two monitoring stations, which may be attributable to a different urban context in which the stations are located. The dissonance between a purely residential context (Rubino station) and another characterised by the co-presence of residential buildings and industries of various kinds (Lingotto station) leads to the need to consider a greater contribution to the calculation of the concentrations emitted in an industrial/residential context due to a greater presence of industrial chimneys but also to more intense motorised vehicle transport. The analysis of the ratio between nitrogen oxides and tropospheric ozone confirmed that, as O_3_ concentration increases, there is a consequent reduction of NO_x_ concentration, due to the chemical reactions of the photo-stationary cycle that takes place between the two species. This work highlighted that the use of an empirical formulation for the estimation of [NO_x_] to [NO_2_] conversion rate could in principle be adopted. However, the application of empirical models for the preliminary estimation of [NO_x_] conversion to [NO_2_] cannot replace advanced models and should be, in principle, restricted to a limited area and a limited range of NO_x_ concentrations.

## Introduction

The sudden changes in lifestyles, the continuous growth of the world population, the progress of technologies applied to combustion, industrial and agricultural processes, and the indiscriminate use of the territory cause a continuous evolution of the characteristics of atmospheric pollution, which need to be understood in order to intervene with effective measures. Thorough knowledge of the behaviour of certain chemical compounds generating atmospheric pollution represents the key point for air quality improvement. Such knowledge is the basis for the planning of specific interventions that aim to reduce possible repercussions on human health and ecosystems (Ravina et al. 2019). Nitrogen oxides (NO_x_) and ozone are among the most important pollutants contributing to the worsening of air quality in urban areas. Nitrogen oxides are classified according to the oxidation state of the nitrogen. The large quantity of diatomic nitrogen present in the atmosphere (N_2_) undergoes a dissociation process when it comes into contact with an O radical, according to the reaction described by the Zel’dovic mechanism (Zeldovich 1946), giving rise to the formation of NO_x_. Of the seven nitrogen oxides, the most dangerous and important ones in the atmosphere are nitrogen monoxide (NO) and nitrogen dioxide (NO_2_). Nitrogen monoxide (NO) is a colourless gas; the limit value for the 8-h working exposure is 5 ppm. The exposure to a concentration of 200 to 700 ppm can be fatal for humans. Nitrogen dioxide (NO_2_) is yellowish-brown in colour; the limit value for 8-h working exposure is 5 ppm. Long exposure times at concentrations of 100 ppm can lead to fatal consequences. For O_3_, international limits are between 0.05 ppm for the short-term exposure and 0.3 ppm for the long-term exposure (American Conference of Governmental Industrial Hygienists (ACGIH; U.S. National Institute for Occupational Safety and Health (NIOSH)). The health-based limit concentrations in the ambient air in Europe, as established by Directive 2008/50/EU (European Union), are 40 μg/m^3^ for NO_2_ (yearly mean) and 120 μg/m^3^ for O_3_ (maximum daily 8-h mean). Emission trends, formation, and fate of NO_x_ and O_3_ have been extensively investigated in the last decades (Ravina et al. 2020a, 2020b). The main anthropogenic sources of NO_x_ are identified in motor vehicles and stationary sources such as power stations and industries. From 1990 to 2018, according to the data provided by the Italian Institute on Protection and Environmental Research, total emissions of NO_x_ in Italy showed a reduction of about 68% (Italian Institute on Protection and Environmental Research 2018). Italy reached the objectives for 2010 identified by the National Directive on Emission Limits, which defined the target value of 990 Gg (European Union 2001). The revised European Directive on Emission Ceilings set a target for Italy of 35% reduction against 2005 emissions in 2030 (European Union 2016). Despite the positive trend, reducing the presence of NO_x_ and O_3_ still represents a challenge for administrations (Magaril et al. 2017).

The authors examined the formation of O_3_ and NO_x_ in the urban environment and found that urban NO_2_, NO_x_ and O_3_ concentrations are closely correlated. A non-linear relationship exists between NO_2_ and NO_x_ (Trebs et al. 2012). It is well known that NO_x_ acts as a key catalyst in the formation of tropospheric ozone (Crutzen and Lelieveld 2001). The presence of nitrogen compounds together with volatile organic substances, catalysed by solar radiation and oxidising compounds such as OH- hydroxyl radicals, generate the end products of photochemical smog such as ozone O_3_ and nitrate-diperoxy-acetyl (PAN). The steady-state concentration of O_3_ depends on the concentrations of NO and NO_2_. If NO_2_ concentrations increase, the ozone concentrations level off at a higher value, although they do not change over time (Seinfeld and Pandis 2016). The presence of HO_2_ peroxydril radicals, which are produced by reactions between volatile organic compounds and OH-oxydril radicals, causes them to react with nitrogen compounds, promoting a second phase of the NO_x_ cycle, producing more NO_2_ and increasing O_3_ concentrations accordingly. Although at present the mechanisms of photo-chemical reactions are well understood, concentration trends and relationships among different players in the urban environment are influenced by a series of factors, the result of which is still sometimes difficult to predict (Degraeuwe et al. 2017). Meteorological factors (temperature, radiation, wind conditions, air humidity) are the main drivers of reactions equilibrium. Generally, in winter, high NO_2_ episodes can be associated to very low wind speeds, temperature inversions and a shallow, stable boundary layer. Summer episodes of NO_2_ are associated with ozone episodes, that is with hot, still, sunny days. Additional research efforts must then be addressed to the joint investigation, in form of monitoring and modelling analyses, of the natural and anthropic factors affecting photo-chemical reactions. This work, based on the existing studies on the formation and reaction of NO_x_ in the atmosphere, analysed the historical series of NO_x_, NO_2_, NO, and O_3_ concentrations in the period 2015–2019 by two monitoring stations (Lingotto and Rubino) of the Regional Air Quality Monitoring System of the Piedmont Region, both located in the city of Turin, NW Italy. The study examined the distribution of NO_x_, NO_2_, and O_3_ concentrations in different time bands. The relationships between different components were analysed, investigating the factors that affect the dispersion of the validated data. A set of empirical equations was then elaborated for the determination of NO_2_ concentration values as a function of NO_x_ and temperature, with the aim of developing site-specific forecasting relationships to be applied to pollutant dispersion modelling. The obtained equations were subsequently compared with similar existing formulations developed in other urban areas worldwide. The final objective of this study was to evaluate the feasibility and limits of developing and applying a simplified empirical model for NO_x_ to NO_2_ concentration conversion. The deeper knowledge of conversion mechanisms is of basic importance for the evolution of local air quality planning strategies, as different pollution sources contribute differently in terms of quali-quantitative characteristics of the emissions. Such analysis could also provide relevant support to the study of particular emission scenarios, as occurred during the COVID-19 sanitary emergency when the emission characteristics of different sources varied consistently if compared to ordinary conditions (Marinello et al. 2020).

This manuscript is organized as follows. In the “State of the art” section, an analysis of the state of the art on NO_x_/NO_2_/O_3_ studies and relationships is reported. Data analysis methodology of the present study is reported in the “Methodology” section. Results are presented in the “Results” section and discussed in the “Discussions” section.

## State of the art

### NO_x_, NO_2_, and O_3_ concentration relationships

The presence of nitrogen compounds together with volatile organic substances, catalysed by solar radiation and oxidising compounds such as OH hydroxyl radicals, generates the end products of photochemical smog such as ozone O_3_ and peroxyacyl nitrates (PAN). Ozone, which in the stratosphere is essential for shielding the passage of ultraviolet radiation, is harmful and toxic in the troposphere. The reactions involving nitrogen compounds and O_3_ in the photochemical cycle are reported in Eqs. 1 to 3.

The three reactions are practically cyclic: reaction II produces ozone and reaction III destroys it. The steady-state O_3_ concentration depends on NO and NO_2_ concentrations. If NO and NO_2_ concentrations increase, ozone concentrations do not change over time, but level off at a higher value. This process is known as the photo stationary cycle.

1 $$\begin{array}{cc}I& N{O}_{2} \to NO+O\end{array}$$

2 $$\begin{array}{cc}II& O+ {O}_{2}+R \to {O}_{3}+R\end{array}$$

3 $$\begin{array}{cc}III& {O}_{3}+NO \to N{O}_{2}+ {O}_{2}\end{array}$$

Investigation of the photo-stationary reactions began in the early sixties with the seminal work of Leighton (Leighton 2014). In 1993, Bower et al. (Bower et al. 1993) summarised the chemistry of urban NO_x_ in eight reactions with NO_2_, NO_x_, and O_3_, light (λ ≤ 420 nm), third body M, hydrocarbon RH, radical R*, peroxide radical RO_2_, radical RO, and finally, O_2_ for the slow three-body reaction with NO. The main chemical sinks for NO_2_ include reactions with OH, forming HNO_3_ during the day, and with O_3_ at night. Regarding the O_3_/NO_x_ ratio in the short term in urban areas, the main factor identified was the removal of O_3_ from emitted NO_x_, which is a dominant loss process for O_3_ whenever local NO concentrations exceed 35 ppb. A parameter to be taken into account when analysing concentrations of nitrogen compounds is the total oxidant OX (NO_2_ + O_3_), which is characterised by lower values at night and higher values during the day. In the warmer months of the year, an increase in oxidant concentrations is observed, peaking in the mid-afternoon, due to the oxidation of NO by peroxide radicals with a consequent increase in O_3_ concentrations. The [NO_2_]/[NO_x_] ratio is an important indicator of the state of the photo-catalytic equilibrium. Different studies analysed this ratio in urban and rural environments. Bower et al. (1993) observed that, overall, the [NO_2_]/[NO_x_] yield varied from 0.17 to 0.5 at the kerbside, 0.47 to 0.59 at urban background sites, and about 0.85 at rural stations. This reflected the dilution of NO_x_ as travel distance increases and hence the greater availability of O_3_. Important confirmations were recently given by the ESCAPE study (European Study of Cohorts for Air Pollution Effects). In this study, the spatial variation of NO_2_ and NO_x_ concentrations between and within 36 study areas across Europe was analysed. The results confirmed that [NO_2_]/[NO_x_] concentration ratios were higher at urban background sites. Variability was in general high between the street and urban background (Table 1). This and other studies confirm that the increased [NO_2_]/[NO_x_] ratios in urban areas in Europe are related to the increased NO_2_ emissions of road traffic sources (Degraeuwe et al. 2016; Kurtenbach et al. 2016). Primary NO_2_ emissions are mainly due to diesel-fuelled vehicles (Anttila et al. 2011; Carslaw et al. 2011). In addition, exhaust gas treatment devices (oxidation catalysts) used for reducing particulate matter emissions by diesel vehicles contribute to an increasing fraction of primary NO_2_ in NO_x_ (Williams and Carslaw 2011).

These findings show that air quality planning strategies should give privilege to actions that focus on the traffic sector (Borrego et al. 2012). Considering temporal variability, a study conducted in Delhi, India, focused on a short period in 2012. Large variations were observed in the NO (<1 ppbv to a peak of 295 ppbv), NO_2_ (<2–47 ppbv) and O_3_ (4–95 ppbv) mixing ratios, all of which showed strong diurnal variation (Chate et al. 2014). Kasparoglu et al. (2018) also analysed the continuous measurements of the hourly O_3_, NO and NO_2_ concentrations at 7 rural and 15 urban sites in the Marmara Region of Turkey during the period from March 2013 to April 2016. The results showed an opposite behaviour among O_3_ and NO_x_ in both rural and urban sites. Other analyses showed that at sites with low O_3_ concentrations and abundant NO_x_ concentrations, the [NO_2_]/[NO_x_] ratio was about 0.4 over a fairly wide range of NO_x_. The work of Kallend (1995) focused on the dispersion of NO_x_ over large distances and the possible causes of oxidant production at ground level and O_3_ generation in the troposphere. Plume monitoring was carried out over distances of up to approximately 100 km, with the aid of special tracers so that the chemical evolution of individual plumes could be correlated with their emission points. The authors concluded that in plumes, NO_x_ can remain unoxidized for long travel times and long distances when dispersion is slow and precursor mixing in the plume is limited. Janssen et al. (1988) measured the [NO_2_]/[NO_x_] ratio as a function of downwind plume travel distance. They expressed this ratio in terms of O_3_ concentration, wind speed, and downwind distance. However, the practical application in real plumes cannot be separated from the parameterization of weather conditions.

### Analytical and empirical models

In recent years, several methods have been proposed for evaluating the amount of NO_2_ that is formed from NO. These include total conversion (U.S. Government Printing Office: Washington, D.C. 1996), the ambient ratio method (ARM) (Chu and Meyer 1991), the ozone-limiting method (OLM) (Cole and Summerhays 1979), and the plume volume molar ratio (PVLM) (Hanrahan 1999). This latter is still one of the most employed in dispersion modelling, as it better simulates the NO-to-NO_2_ conversion chemistry during plume expansion. More recently, NO_x_ chemistry schemes were also implemented in Lagrangian dispersion models. For example, Oettl and Uhrner (2011) implemented a hybrid scheme in the GRAL-C dispersion model, where the transport and turbulent diffusion of primary species such as NO and NO_2_ were treated in a Lagrangian framework while those of O_3_ were treated in a Eulerian framework. Despite the results achieved, the correct estimation of NO_x_ to NO_2_ conversion, especially in urban areas and roadways, is still a challenge of dispersion modelling. A study conducted in Las Vegas, USA (Kimbrough et al. 2017), showed that under high O_3_ conditions, NO_x_ chemistry is driving the [NO_2_]/[NO_x_] ratios, whereas under low O_3_ conditions, atmospheric mixing is the driving factor. This aspect is not taken into account by the chemical formulations implemented in dispersion models. Thus, ambient measurements must be analysed and processed. As a result, empirical relationships can be derived and applied to a restricted spatial or temporal frame. The considerable advantage of determining NO_2_ concentrations in relation to NO_x_ using an empirical polynomial function is that it provides a rapid estimate of NO_2_ concentrations that can also be useful in assessing possible future scenarios. In the following, four different empirical formulations are reported. These formulations were employed for comparison in the present study. The reported models are all in dimensional form; thus, users must employ the correct units at the time of their application.

#### Derwent-Middleton curve

The study conducted by Derwent and Middleton (Derwent and Middleton 1996) analysed hourly ppb concentrations of NO_x_, sorting the considerable amount of data into 10 classes per ppb together with the NO and NO_2_ concentration values. The concentration values were then averaged for each individual bin to derive a curve fit to the upper limit of the NO_x_ bin. The relationship used is as follows:

4 $$\left[{NO}_{2}\right]=2.166- \left[{NO}_{x}\right]\left(1.236-3.348 {A}_{10}+1.933 {{A}_{10}}^{2}-0.326 {{A}_{10}}^{3}\right),$$

where the square brackets indicate the hourly average concentration in ppb, and A_10_ = log([NO_x_]). This function was applied in the range 9.0 ppb < [NO_x_] < 1141.5 ppb. Below 9.0 ppb of NO_x_, the efficiency of [NO_2_]/[NO_x_] ratio was limited to 0.723. Above 1141.5 ppb of NO_x_, [NO_2_]/[NO_x_] ratio was limited to 0.25.

#### Dixon-Middleton-Derwent polynomials

The Dixon et al. study (2001), on the basis of a larger amount of data in the curve fit than that used by Derwent and Middleton (1996), investigated the validity of new empirical relationships at several sites. The point of significant difference from previous studies was to consider the ratio between NO_2_ and NO_x_ concentrations as a dimensionless yield:

5 $$Y=\frac{\left[{NO}_2\right]}{\left[{NO}_x\right]},$$

where [NO_x_] = [NO] + [NO_2_] (ppb), and 0 ≤ Y ≤ 1 (dimensionless). The monitoring data were sorted by increasing NO_x_ concentrations in bins 10 ppb. The study defines NO_x_ concentrations as the only independent variable, thus assuming a significant simplification of dispersion and chemical processes to determine NO_2_ yield. The resulting function for defining the yield is reported in Eq. 6:

6 $${Y}_{2}= A+B{A}_{10}+C{{A}_{10}}^{2}+D{{A}_{10}}^{3}+E{{A}_{10}}^{4}.$$

At urban sites, dimensionless parameters A, B, C, and E of the function take on the following values:

7 $${Y}_{2}= - 3.08308+7.472477 {A}_{10}-5.11636 {{A}_{10}}^{2}+1.381938 {{A}_{10}}^{3}- 0.12919 {{A}_{10}}^{4}.$$

The authors indicated a root mean square error (RMSE) of about 30 - 50% of the annual mean concentrations. A study carried out by Carslaw et al. (2001) focused on hourly NO_2_ curves as a function of NO_x_. The authors theorised that there are three distinct parts to the curve produced by averaging each bin according to the Derwent and Middleton method: a region in excess of O_3_ (where NO_2_ increases rapidly with NO_x_ at low values), a region with limited O_3_ (where NO_2_ increases slowly with respect to NO_x_), and a peak related to a winter episode where NO_2_ increases again, probably due to the three-body NO + O_2_ reaction.

#### Clapp

The study conducted by Clapp (2001) investigated the influence of solar radiation on O_3_, NO and NO_2_ concentrations as a function of NO_x_ in rural and urban environments. Variations in O_3_ concentration at the global level influence O_3_ and NO_2_ concentrations at the local level, and consequently, the response of NO_2_ to NO_x_ emission reduction is highly non-linear. In relation to the total concentrations of oxidants OX (Eq. 8, ppb unit), Clapp studied how this varies with NO_x_ concentrations.

8 $$\left[OX\right]= \left[{O}_{3}\right]+ \left[{NO}_{2}\right].$$

The plot of the OX parameter as a function of NO_x_ showed a linear trend characterised by a positive intercept, indicating a value that is a function of the region under observation, and an upward slope representing local or primary NO_x_ emissions. On the basis of this evidence, the authors identified two main contributions to diurnal concentrations of total oxidants: a regional (or background) contribution that is close to O_3_ and largely independent of NO_x_, and a local contribution that is correlated with (i) the primary contribution of local NO_x_ emissions to local NO_2_, (ii) local oxidation of NO to NO_2_ by O_2_, and (iii) local emissions of some species, such as HONO that may contribute to the conversion of NO to NO_2_. The study yielded the following relationship (ppb unit):

9 $$\left[OX\right]=0.104 \left[{NO}_{x}\right]+31.1$$

In the regulatory field, this study could be used to introduce more restrictive limit values for the species under study, at certain times of the year, when significant variations in regional contribution due to photochemical episodes are expected.

Jenkin (2004a) also reported an idealised equation to determine NO_2_ concentrations as a function of NO_x_, given the total concentration of oxidants OX (Eq. 10).

10 $$\left[{NO}_{2}\right]= \frac{-Z \pm \sqrt{{Z}^{2}-4\left[{NO}_{x}\right] \left[OX\right]}}{2},$$

where

11 $$Z= -\left(\left[{NO}_{x}\right]+ \left[OX\right]+ \frac{J}{K} \right) \left[ppb\right],$$

In Eq. 11, J is the NO_2_ photolysis rate [s^-1^] and K is the chemical reaction rate constant for NO + O_3_ [ppb^-1^ s^-1^]. The author used annual mean values equal to J = 2.2 ∙ 10^-3^ s^-1^ and K = 3.7 ∙ 10^-4^ ppb^-1^ s^-1^, from which a J/K ratio of 5.9459 ppb derives.

The importance of the parameter OX is highlighted in Clapp (2001) where monitoring data were used to define the trend of OX concentrations in relation to NO_x_ concentrations, resulting in straight graphs with Eq. 12 (ppb unit):

12 $$\left[OX\right]=A \left[{NO}_{x}\right]+B,$$

where slope A represents the gradual increase in local OX as NO_x_ increases, and intercept B, which is a constant and NO_x_-independent OX value, represents the regional oxidant concentration. The results obtained from the study show that the values of the slopes vary according to the region under study, with most of the values falling in the range 0.1–0.2, unlike the values of the intercepts, which are very similar and almost all fall in the range 33 ± 1 ppb. Jenkin’s method made possible, on the basis of the concentrations of the regional oxidant and the local oxidant, to plot the annual average of NO_2_ against NO_x_, and could be used to predict possible increases in the regional oxidant in relation to climate change.

#### Jenkin and Stedman et al.

The Jenkin study (2004a) looked at estimating the annual average NO_2_ and the annual average O_3_ as a function of the annual average local NO_x_. The sites under consideration relied on the monitoring of the species under study so that the OX parameter could immediately be determined. By defining the concentrations of the regional oxidant (intercept B) and the local oxidant (slope A), it was possible to divide the sites analysed into two groups based on the proximity of the measuring station to nearby roads. Equation 13 was used to include data from sites close to roads. Equation 14 was used for sites located at a certain distance from roads.

13 $$\frac{\left[{NO}_{2}\right]}{\left[OX\right]}= {f}_{1}\left({NO}_{x}\right)=8.962 \times {10}^{-2}+1.474 \times {10}^{-2} x-1.290 \times {10}^{-4} {x}^{2}+5.527\times {10}^{-7} {x}^{3}-8.906 \times {10}^{-10} {x}^{4},$$

14 $$\frac{\left[{NO}_{2}\right]}{\left[OX\right]}= {f}_{2}\left({NO}_{x}\right)=1.015 \times {10}^{-1}+1.367 \times {10}^{-2} x-6.127 \times {10}^{-5} {x}^{2}-4.464\times {10}^{-8} {x}^{3},$$

where x is equal to [NO_x_] (ppb unit);

Jenkin’s formulation for sites that are at a fair distance from roads is in agreement with another formulation from a study by Stedman et al. (2001, Eq. 15).

15 $$\left[{NO}_{2}\right]= \chi {\left(\left[{NO}_{x}\right]\right)}^{0.6887},$$

where the factor χ (dimensionless) varies according to the location of the site (usually 1.58–1.76).

This method was further analysed by Jenkin (2004b), considering hourly measurements, finding that under conditions of low NO_x_ concentrations, typically at night during the summer period, NO_2_ was only 25% of the OX. Conversely, under conditions of high NO_x_ concentrations, typically during daytime hours of the winter period, NO_2_ constituted most of the OX. Based on the reported information, it is suggested that the variation in NO_2_ and NO_x_ concentrations depending on the time of day and seasonality of the measurements should be taken into account for the assessment of limit concentrations in relation to environmental quality standards.

## Methodology

This work involved the analysis of the time series of hourly averages of nitrogen species concentrations. The data used to study the relationships between nitrogen species in the tropospheric domain were obtained from the air quality monitoring system of the Piedmont Region, Italy (Piedmont Region Environmental Protection Agency).

### Description of the area

**Fig. 1:**
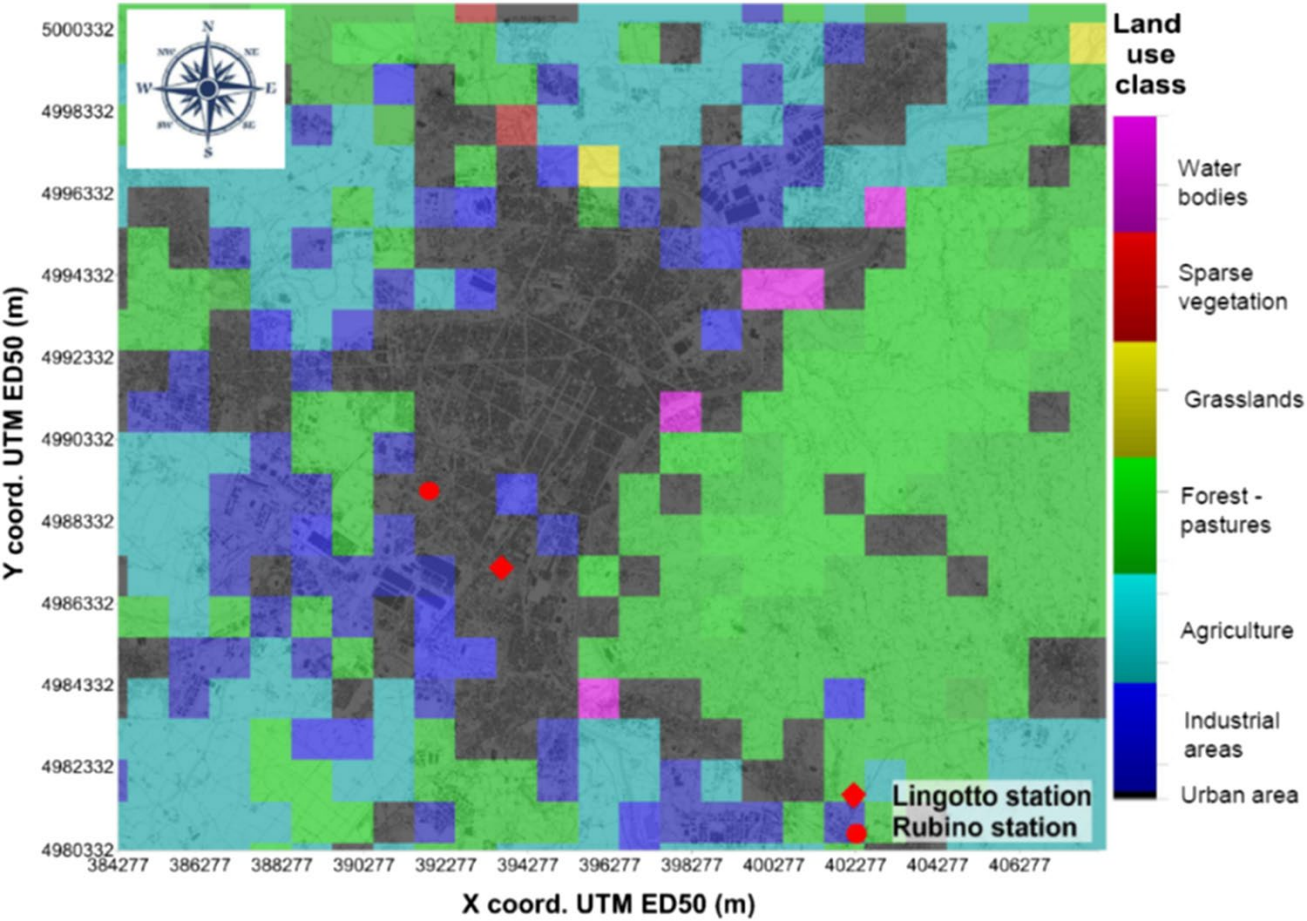
Location of the monitoring stations and land use classification of the area.

**Fig. 2:**
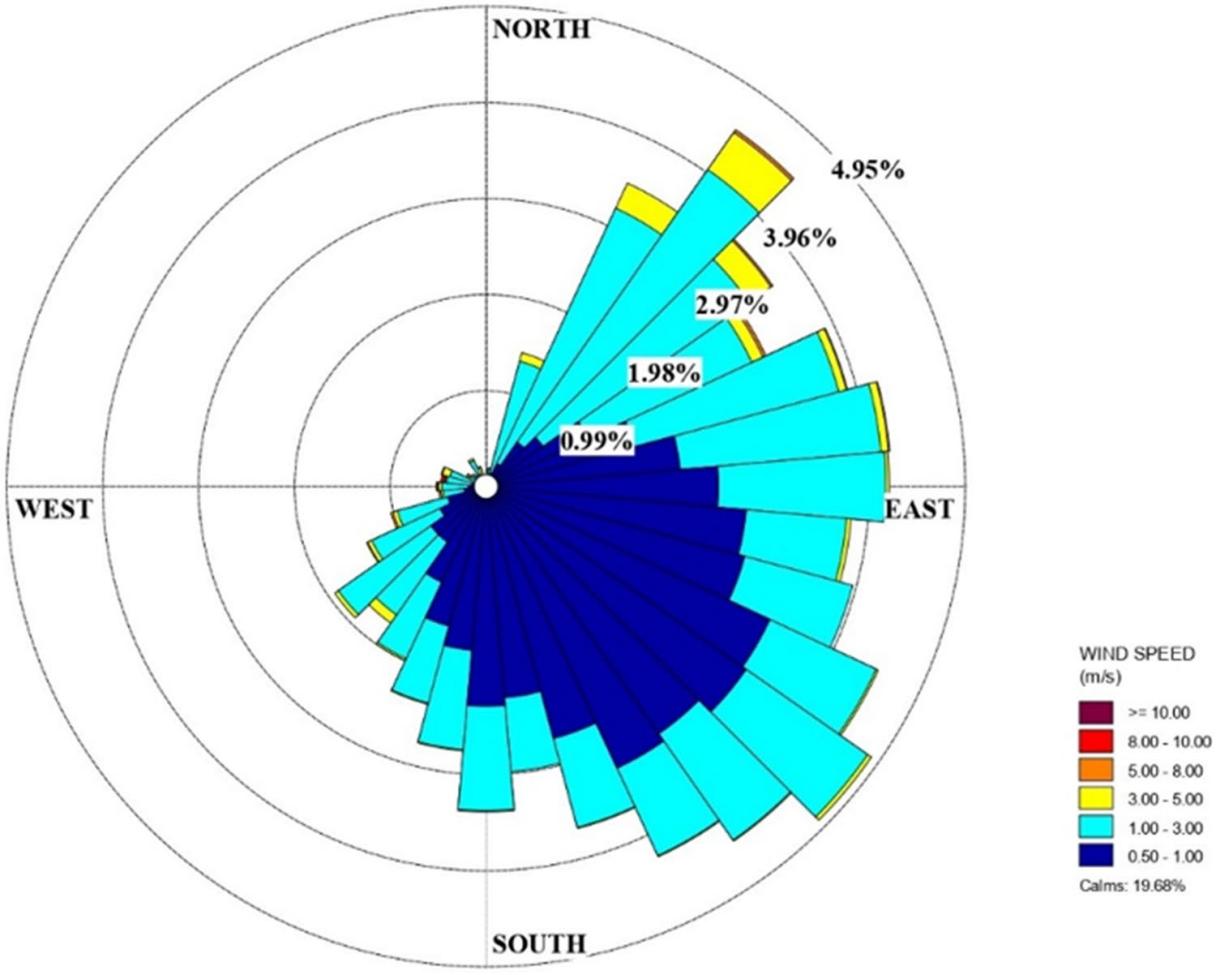
Wind distribution for the year 2019 in Turin

Turin, the capital of the Piedmont region, is a highly industrialized city and densely populated metropolitan area, enjoying a humid subtropical climate. Turin is the fourth largest city in Italy, with around 870, 000 inhabitants and a population density of 6, 730 inhabitants/km^2^ (Figure 1). It is located in the Po Valley, northern Italy. Due to the high anthropogenic activity and its geophysical conformation, the Po valley is presently one of the most polluted areas in Europe (Ravina et al. 2017). The city suffers from low dissolution of pollutants, since it is surrounded by the Alps and hills in the North, West and East. This area is characterized by low winds, in particular during summer and winter. The average value of wind speed from 1990 to 2004 was 0.9 m/s. The wind distribution of the year 2019 is reported in Figure 2. The average annual number of wind calm days was equal to 75 (Piedmont Regional Environmental Agency 2007). During the cold season, pollutant dispersion is mainly regulated by local breeze regimens and soil heat-induced turbulence, which is minimum from December to February. Precipitations are minimum in January.

According to the data provided by the Regional Emission Inventory (Piedmont Region 2015), total NO_x_ emission in Turin in 2015 was 7, 671 t, with the following contribution of emission source typologies: residential combustion, 13%; industrial activities, 14%, road traffic, 71%; and other sources, 2%.

### Data acquisition and processing

The observed concentrations cover the period from 2015 to 2019 at two monitoring stations located in the municipality of Turin, respectively the Turin-Lingotto station and the Turin-Rubino station (Figure 1). Both stations are classified as urban background stations. The instruments used to measure the concentrations of nitrogen oxides at the Torino-Lingotto and Torino-Rubino monitoring stations are the TELEDYNE API 200E and the TELEDYNE API 200A, respectively (Teledyne). Both instruments return an hourly average concentration value using the chemiluminescence measurement method as specified by UNI EN 14211:2012 (UNI EN 2012). These instruments use a cooled photo-multiplier tube (PMT), to detect the amount of light created by the NO and O_3_ reaction in the reaction cell. Measurements are affected by intrinsic noise. To determine how much noise remains, the sample gas flow is periodically diverted directly to the vacuum manifold without passing the reaction cell, performing an auto-zero corrected reading. The concentration of ozone is measured with the TELEDYNE API 400E instrument in both stations. This system is based on the Beer-Lambert law. A 254 nm UV light signal is passed through the sample cell where it is absorbed in proportion to the amount of ozone present. Measurements were validated by the Piedmont Region Environmental Agency. Validation efficiency results of 90%, as required by national and EU regulations. The mean value of NO_2_ concentrations was determined for all validated data in relation to the two measurement stations. The method adopted is the one described in Carslaw et al. (2001) used to determine the annual mean NO_2_ concentration, adapted to the present case study. The average was obtained by sorting the NO_x_ concentrations into frequency classes, with a range of 10 μg/m^3^, and averaging the NO_2_ concentrations for each of the respective classes. The class averages were multiplied by the number of observations in each class. Finally, the average concentration [NO_2_] (μg/m^3^ unit) was calculated by dividing the sum of the product of the averages [NO_2_(i)] and their frequencies F(i) by the number of total observations N_tot_ (Eq. 16):

16 $$\left[{NO}_{2}\right]=\frac{\sum_{i=1}^{n}\left[{NO}_{2}(i)\right] F(i)}{{N}_{tot}}.$$

The analysis was differentiated considering the hot and the cold season. The period from April to September was considered for the hot season and the remaining 6 months of the year for the cold season. With regard to the day-night observation interval, hourly average concentrations from 7 a.m. to 7 p.m. were taken into account for the daytime period and the remaining twelve hours for the nighttime period. The total number of data to be analysed is reported in Table 2. An initial assessment showed that above a value of 500 μg/m3 for NO_x_ concentrations, the number of data decreased significantly. For this reason, it was chosen to work on a concentration range of 0 to 500 μg/m^3^.

Although some of the NO_2_ concentrations in the atmosphere are related to primary NO_2_ emissions, ambient NO_2_ concentrations are mainly attributed to the secondary production of NO_2_ in the atmosphere through photochemical processes. The evolution of nitrogen oxides in relation to ozone concentrations at both monitoring stations was thus analysed. Several studies showed that, due to the chemical interaction of O_3_ with nitrogen oxides NO_x_, NO_2_, and O_3_ concentrations are strongly linked to each other (Clapp 2001). Finally, the relationship between nitrogen species and the temperature was analysed. This phase of the study considered the concentrations of NO_x_ and NO_2_ during the year 2018 only, in relation to the temperatures measured at a meteorological station near the city centre of Turin.

### Empirical relationships

Unlike the study produced by Clapp (2001), which takes into account not only the total concentrations of oxidants present but also the kinetic constant K and the parameter J (also improperly a kinetic constant), the model adopted in this study for the definition of the empirical relationships is based on the work carried out by Derwent and Middleton (1996) and by Dixon et al. (2001). In both studies, the observed concentration values are arranged in ascending order and divided into classes. While Derwent and Middleton defined a polynomial equation that expressed the NO_2_ concentration as a function of the logarithm of the NO_x_ concentration, Dixon elaborated a polynomial function still related to the logarithmic function of the NO_x_ concentration, but which returned a dimensionless value of the [NO_2_]/[NO_x_] ratio. On the basis of these considerations, the polynomial functions were derived on the basis of the average values returned by the division into classes of 10 μg/m3 as a function of the NO_x_ species. Polynomial functions were evaluated with Matlab software polyfit function, which adopts a least-squares approach.

## Results

### Temporal trends

**Fig. 3:**
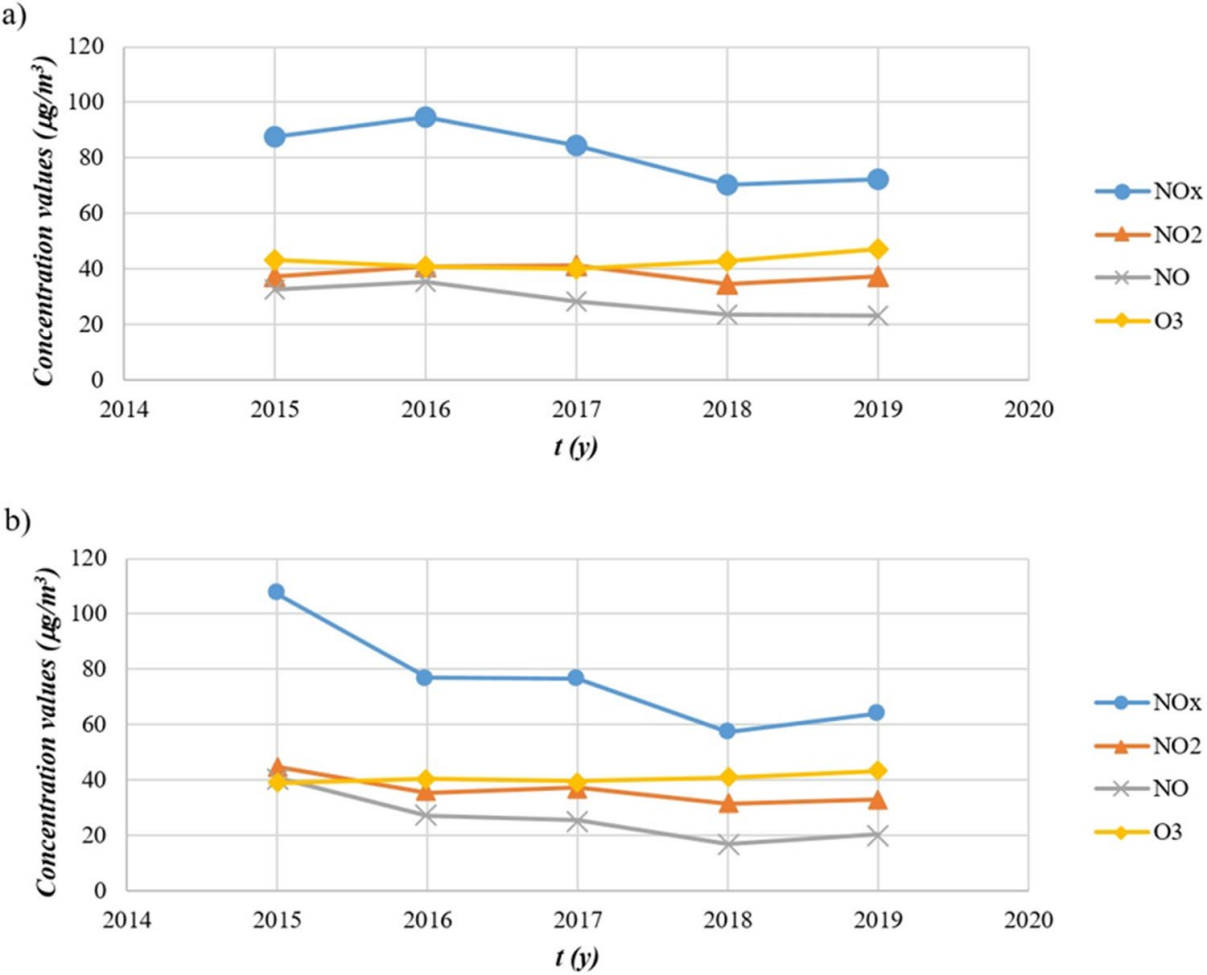
Trend of concentrations during the data collection period in **a** Lingotto station and **b** Rubino station

The trend of the average NO_x_ and O_3_ concentrations derived during the 5 years considered is reported in Figure 3.

Figure 3 shows a downward trend in NO_x_ concentrations for both monitoring stations, with a slight upward trend in 2019. With reference to the legal limit for atmospheric emissions of 40 μg/m^3^ for NO_2_ (Italian Republic 2010), this threshold was exceeded in 2016 and 2017 for the Lingotto station, and in 2015 for the Rubino station. The trends of the other observed chemical species are substantially similar in the two graphs, except for a higher NO_2_ concentration than O_3_ concentration in 2015 for the Rubino station, coinciding with the highest averaged NO_x_ value. In order to match the relationship between NO_2_ concentrations and ground-level ozone, the validated data were divided according to the time of detection and then averaged to report the 24-h trend of the chemical species under study.

**Fig. 4:**
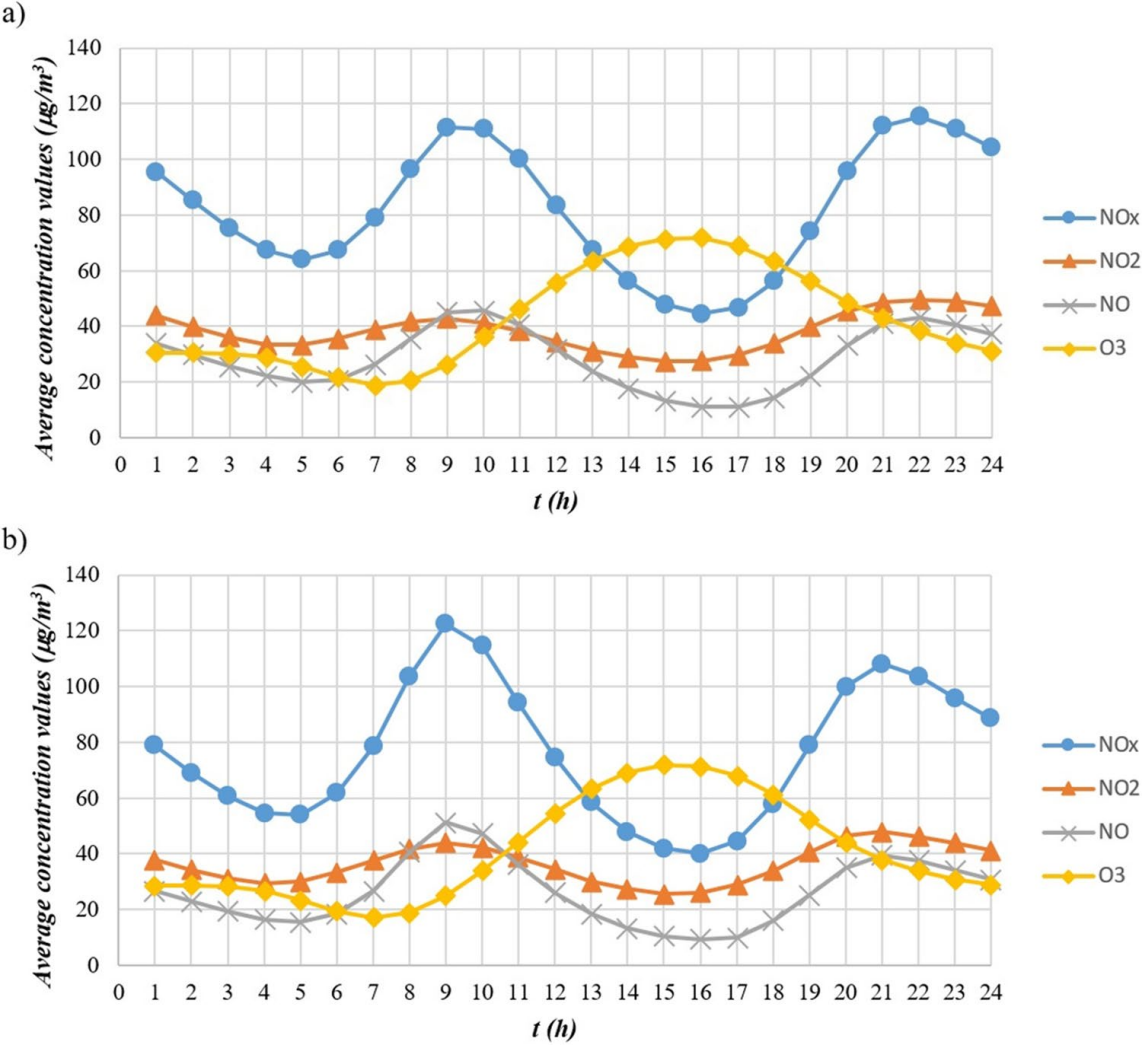
Average concentration values over the day in **a** Lingotto station and **b** Rubino station

In Figure 4, a bimodal trend is evident for NO_x_, with concentration peaks in morning hours, around 9 a.m., and in the evening hours between 9 p.m. and 10 p.m. The close dependence between NO_2_ and O_3_ is confirmed by the concentration trends of the two species. In fact, in both figures, a counter-phase trend of the two curves can be observed, which is more evident in the time band between 10 a.m. and 8 p.m. in which the maximum of O_3_ concentration corresponds to the minimum of NO_2_ concentration. It should also be noted that the maximum O_3_ concentration corresponds to the minimum NO_x_ concentration. This shows that, as the concentration of O_3_ increases, there is a consequent reduction of NO_x_ concentrations. This is due to the chemical reactions between nitrogenous species and tropospheric ozone, belonging to the photo-stationary cycle of ozone formation, which is activated by solar radiation and begins with the photolysis of NO_2_.

**Fig. 5:**
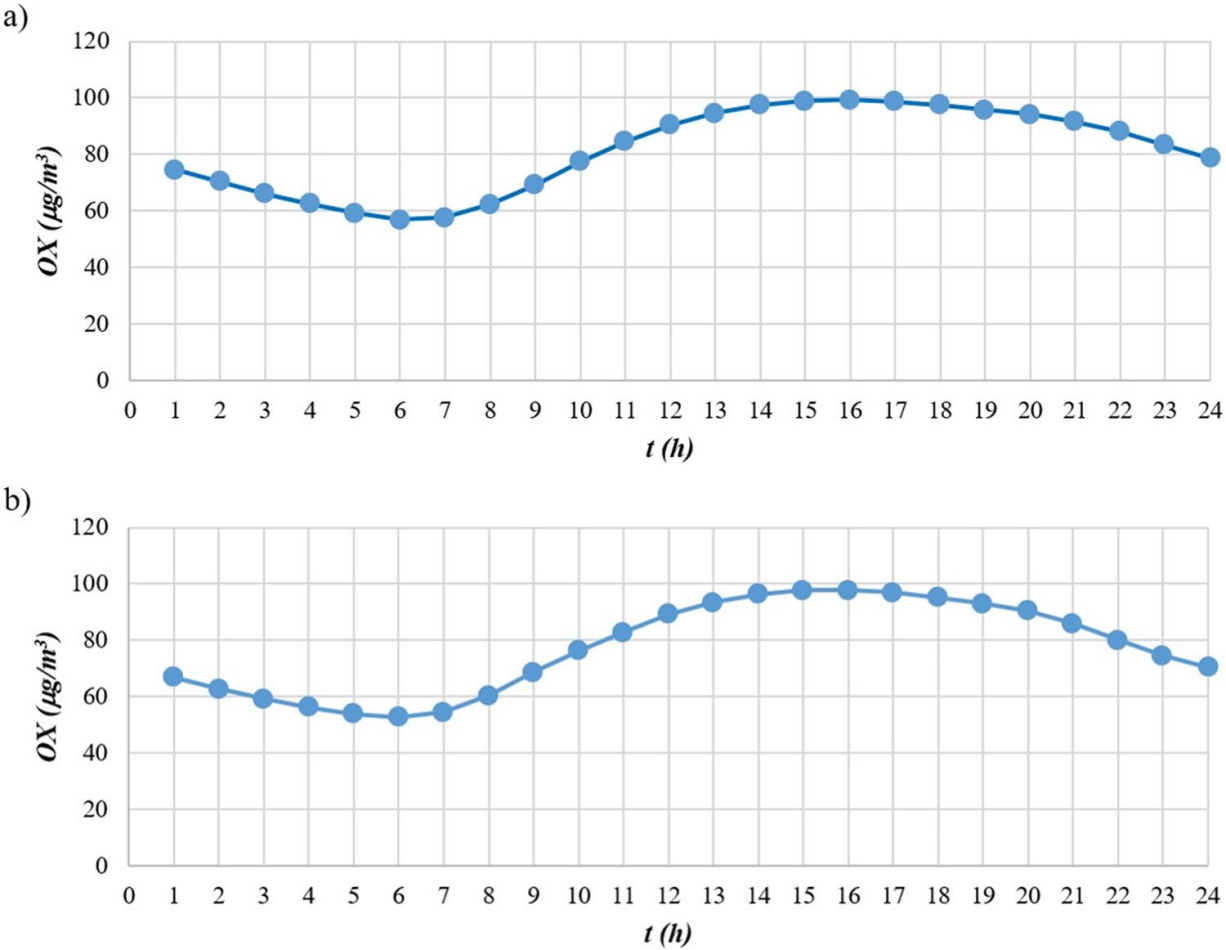
Average OX concentration over the day in **a** Lingotto station and **b** Rubino station

A further parameter that is widely used to assess the interconnections between nitrogen species and tropospheric ozone is the level of photochemical oxidant OX, defined as the sum of O_3_ and NO_2_ concentrations. Several studies have observed that at low NO_x_ concentrations, the main component of OX is O_3_, while NO_2_ is the dominant component of OX under conditions of high NO_x_ concentrations (Jenkin 2004a). In Figure 5, reporting the photochemical oxidant trend for the two monitoring stations, a well-defined concentration trend can be observed, characterised by a minimum point around 7 a.m., followed by a rise in concentrations culminating around 3 p.m., and a gradual decrease until 12 p.m.

### Average concentrations of NO_*2*_, NO_*x*_, and NO (cold/hot, night/day)

**Fig. 6:**
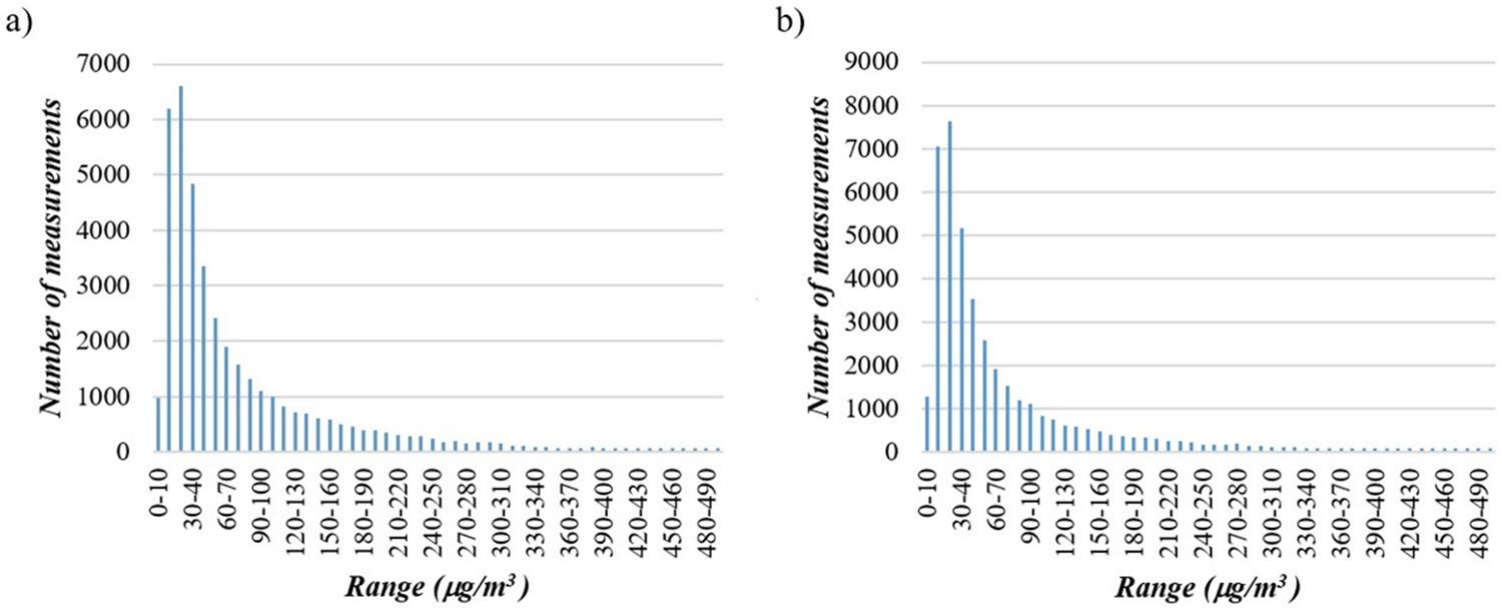
Detection frequency of NO_x_ concentrations: **a** Lingotto and **b** Rubino

Concentration distributions (Figure 6) show a substantially equal trend among the two monitoring stations, with frequency peaks in the intervals between 10 and 20 μg/m3 and 20 and 30 μg/m^3^, followed by a gradual decrease. Marked differences are observed at both stations when comparing the frequency distributions between the hot and cold seasons. Specifically, in the warm season, the data show a marked peak in the intervals between 10 and 20 μg/m^3^ and 20 and 30 μg/m^3^, followed by a rapid decrease. On the contrary, in the cold season, the trend is dissimilar. This can be explained by two factors: the increase of NO_x_ emissions due to residential heating and the meteorological conditions, i.e., less solar radiation dissociating NO_2_ into NO. Lastly, when comparing day and night time intervals, during the daytime the peak is distributed over two intervals, while, at night, the highest frequency is found in the 20–30 μg/m^3^ interval.

### [NO_2_]/[NO_x_] relationship

On the basis of the validated data of NO_x_ and NO_2_ concentrations, scatter plots and NO_x_ vs NO_2_ curves for the data were elaborated.

**Fig. 7:**
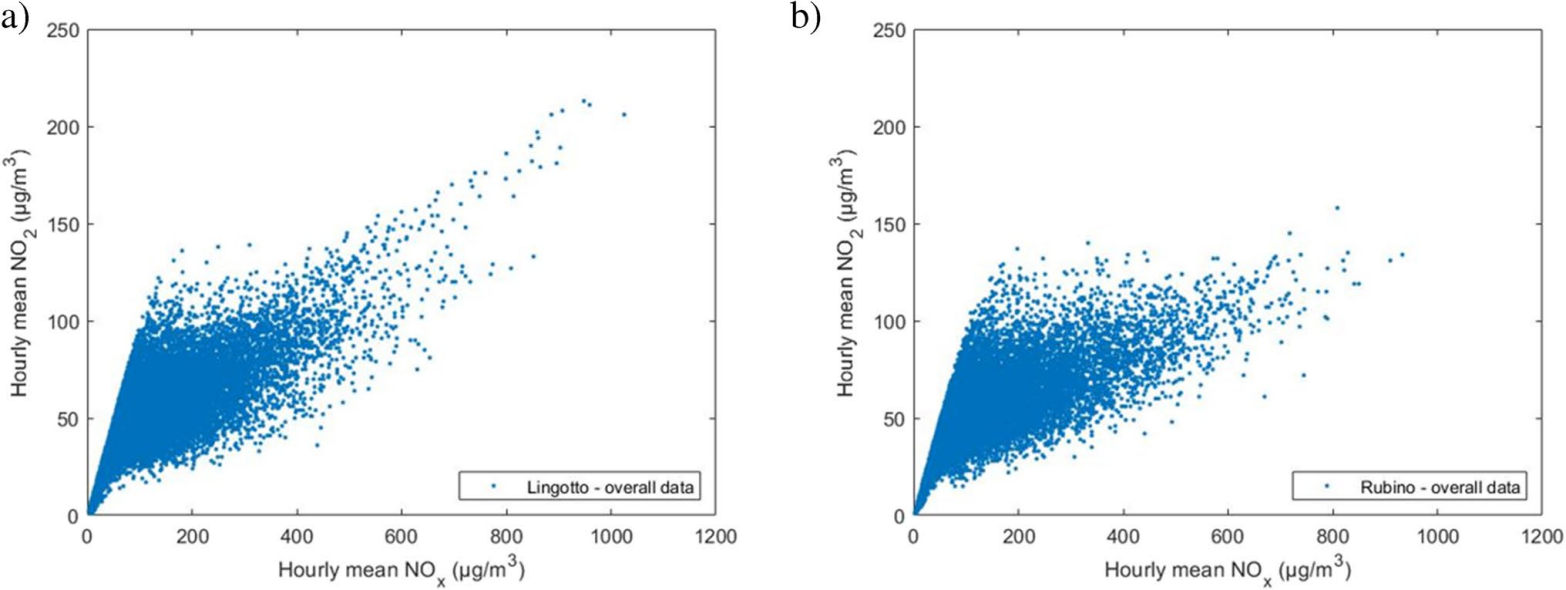
Scatter plot of the overall data for **a** Lingotto station and **b** Rubino station

**Fig. 8:**
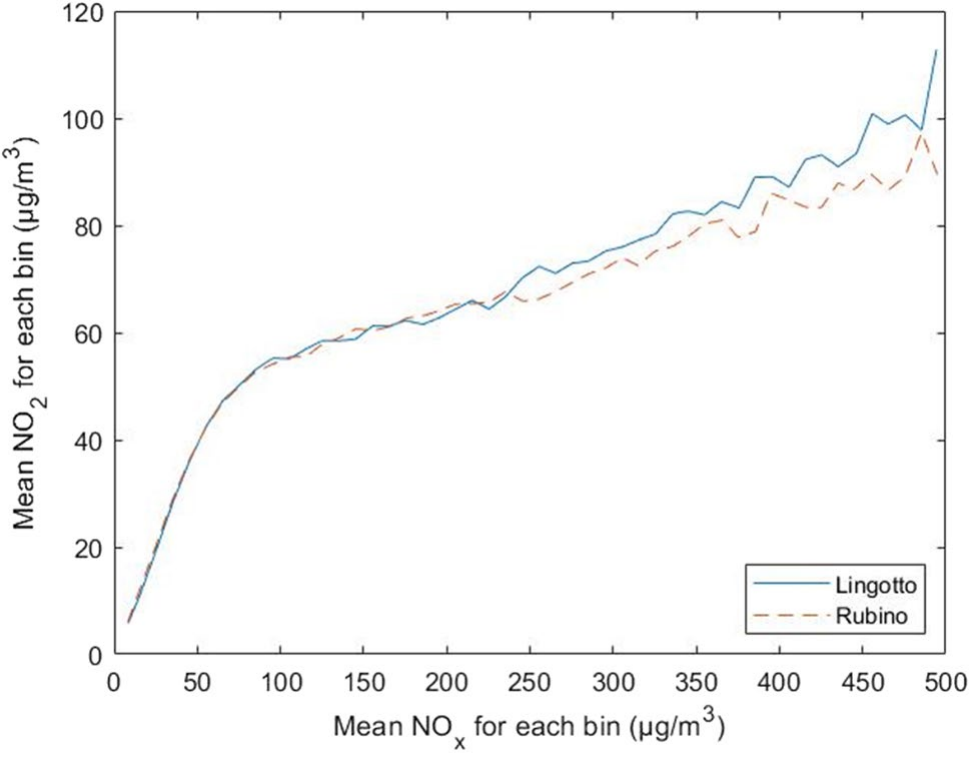
[NO_2_]/[NO_x_] trend at the two monitoring stations

Figure 7 shows an equal distribution of the concentrations between the two measurement stations, except for a tail in the scatter plot of the Lingotto station, included in the range 600 ÷ 1000 μg/m^3^ of NO_x_. This highlights a specific condition in which this particular distribution of concentrations occurs, i.e., low temperatures and absence of solar radiation, but at the same time does not explain why. Given the same boundary conditions, this distribution of points is not also present in the scatter plot relative to the Rubino station. A possible reason can be the presence of more industrial/residential emissions and a more intense road traffic close to the Lingotto station. Recent studies carried out in various urban areas indicate precisely how potential emissions from motor vehicles affect NO_2_ concentrations (Carslaw and Beevers 2005; Kimbrough et al. 2013; Richmond-Bryant et al. 2017). These studies show an increase in the [NO_2_]/[NO_x_] concentration ratio due mainly to emissions from road transport, and attributable mainly to the use of particulate filters in diesel cars. This increase in the [NO_2_]/[NO_x_] ratio as a function of motor vehicle emissions is reflected in Figure 8 where a different concentration trend is observed once the 240 μg/m^3^ NO_x_ value is exceeded, with a higher [NO_2_]/[NO_x_] ratio in the Lingotto station with respect to the Rubino station. This increase in NO_2_ concentrations at the Lingotto station was observed in all the time periods.

**Fig. 9:**
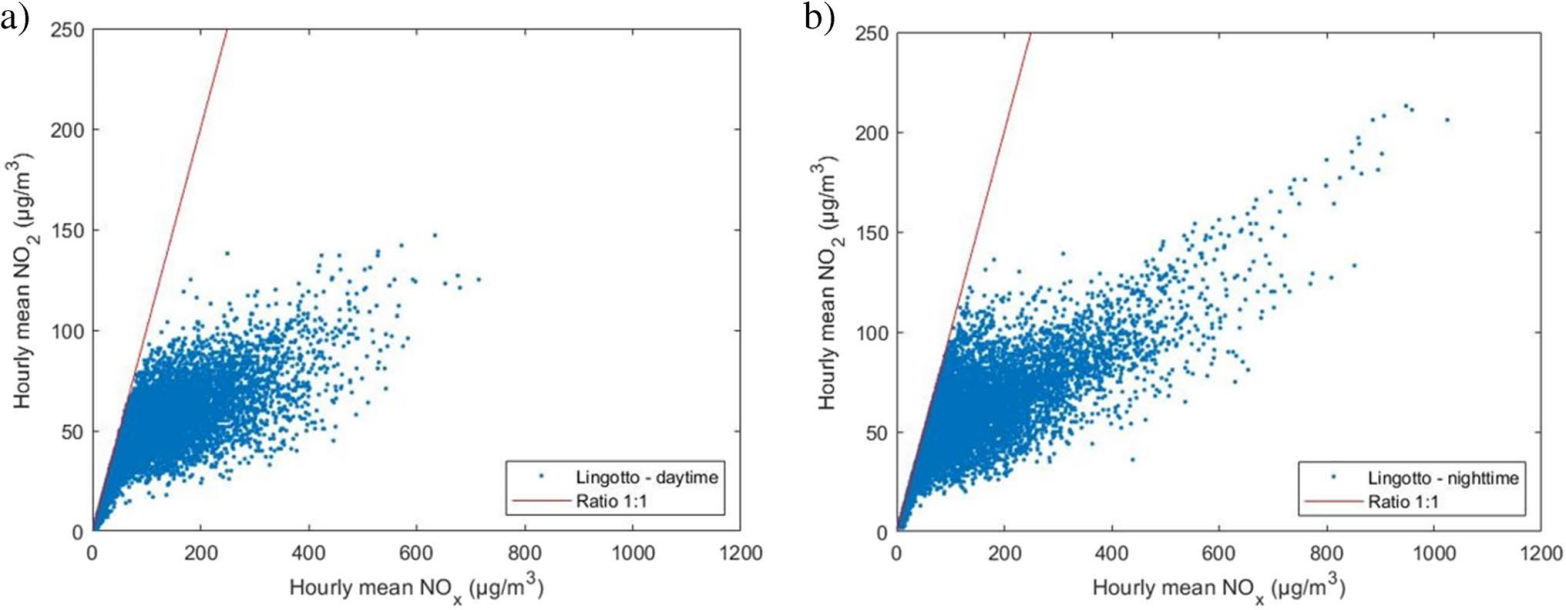
Scatter plot of concentrations observed at the Lingotto station in **a** daytime and **b** nighttime

With regard to the analysis of the daytime and nighttime time bands, the scatter plots reported in Figure 9 show how concentrations are distributed in a similar manner for both stations and emphasize that, unlike the nighttime distributions, the concentration values measured in the presence of light are mainly concentrated below 80 μg/m^3^ for NO_2_. This difference between daytime and night-time distributions is easily explained by the contribution of solar radiation, which dissociates NO_2_ into NO.

In the final part of this work, empirical relationships were derived for the overall dataset and for all the time frames considered in the statistical analysis.

**Fig. 10:**
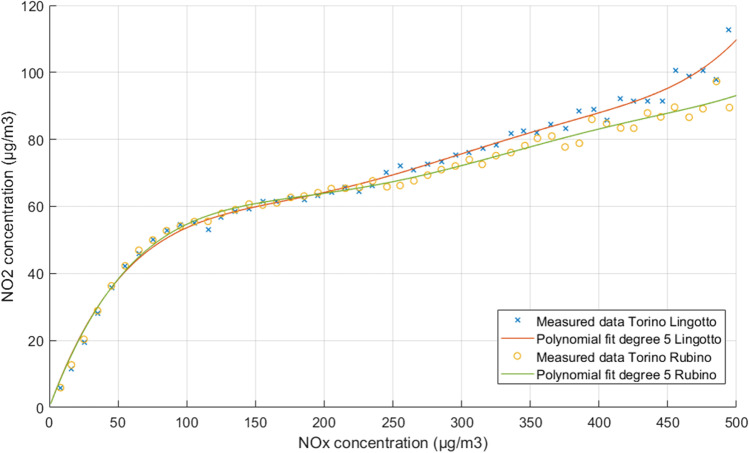
Measured NO_x_ and NO_2_ concentrations in Turin, Italy, and related polynomial fit

Curve fitting was elaborated by using the Matlab software, employing the Polyfit function and searching for the adequate polynomial degree for the fitting of the empirical curve to the input data. The best fitting was selected based on the highest R^2^ and lowest RMSE values. The procedure yielded a polynomial function of degree 5, with the following general equation:

17 $$y=p1\bullet {x}^{5}+p2\bullet {x}^{4}+p3\bullet {x}^{3}+p4\bullet {x}^{2}+p5\bullet x+p6,$$

where y = [NO_2_] and x = [NO_x_] (μg/m^3^ unit). The parameters and stats of the equation obtained are reported in Table 3. Figure 10, which reports the approximation curves of the polynomial function for the Lingotto station and the Rubino station, shows a similar trend between the two functions, with a rapid rise in NO_2_ concentrations in the range 0 ÷ 100 μg/m^3^ of NO_x_, followed by a gentler rise in the curve. It is interesting to note that, in both graphs, there is a slight inflection point in the range of 140–160 μg/m^3^ NO_x_. The reasons of this trend are not clear and should be investigated against additional datasets, to discern if it is due to physical factors, or rather be fictitious due to a high uncertainty affecting the concentration observations. The only difference is found at high concentrations, where the curve for the Lingotto station has an upward tail, while the curve for the Rubino station ends in line with the trend of the function.

### Influence of temperature

**Fig. 11:**
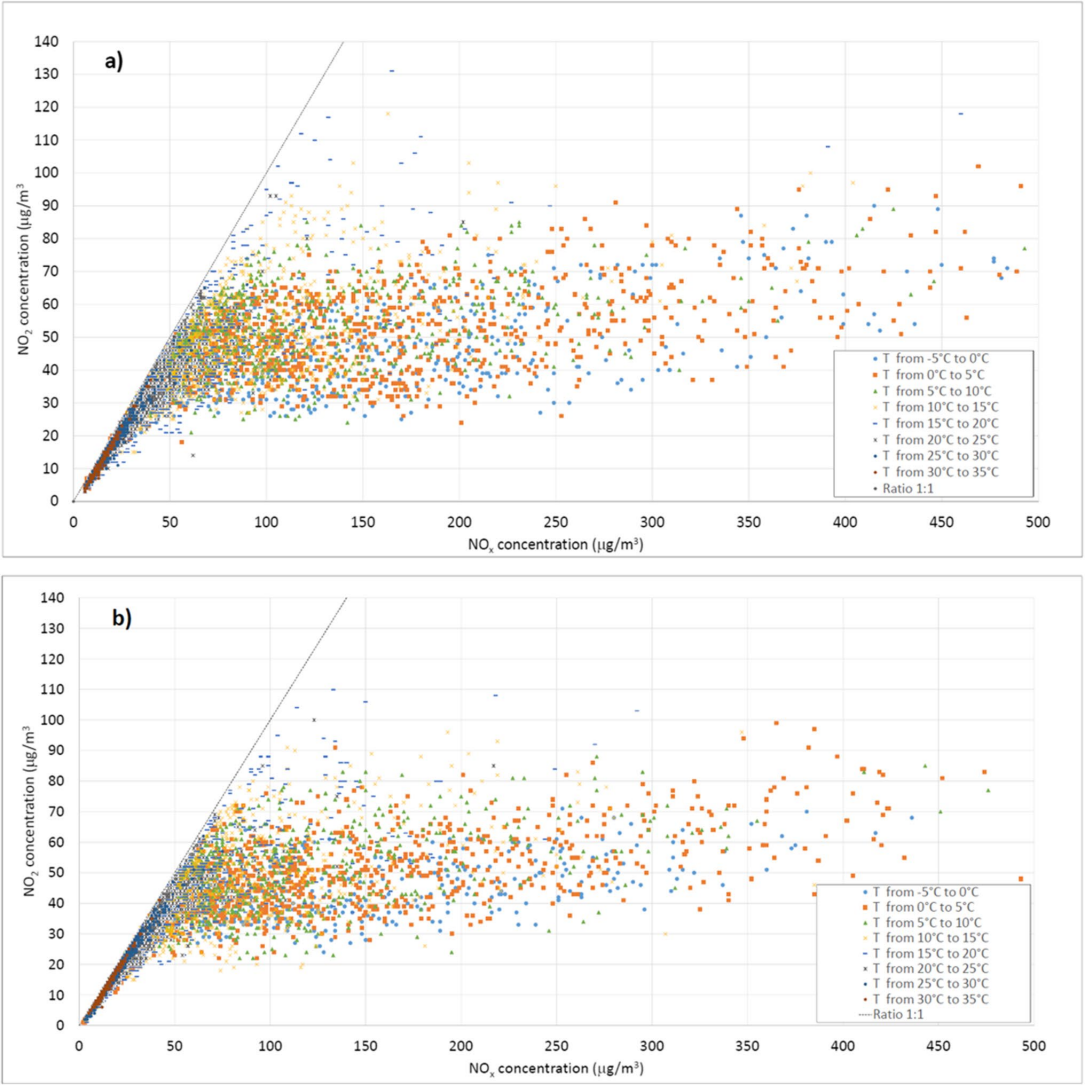
Distribution of concentrations in relation to the different temperature ranges at **a** Lingotto station and **b** Rubino station

The scatter plots of NO_2_ concentrations as a function of NO_x_, classified on the basis of different temperature ranges, are reported in Figure 11.

In Figure 11, it is evident that the range 0 ÷ 100 μg/m^3^ for NO_x_, is characterised by temperature ranges above 20°C, with NO_2_ concentration values increasing as the temperature decreases. In the range 100 ÷ 300 μg/m^3^ for NO_x_, there is a denser distribution that occupies a wider domain of NO_2_ concentrations, ranging from 20 to 80 μg/m^3^, with temperatures mainly between 5 and 20 °C. Above 300 μg/m^3^ of NO_x_, a greater dispersion of the distribution is observed with temperatures not exceeding 10 °C. Finally, the best fit for the distribution of [NO_2_]/[NO_x_] concentration ratios as a function of temperature was investigated. Among the possible solutions, although the application of a polynomial fit yielded the best stats, this relationship was best approximated by applying a sigmoid function in the form of:

18 $$y=\frac{a+b}{c+{e}^{-dx}}$$

**Fig. 12:**
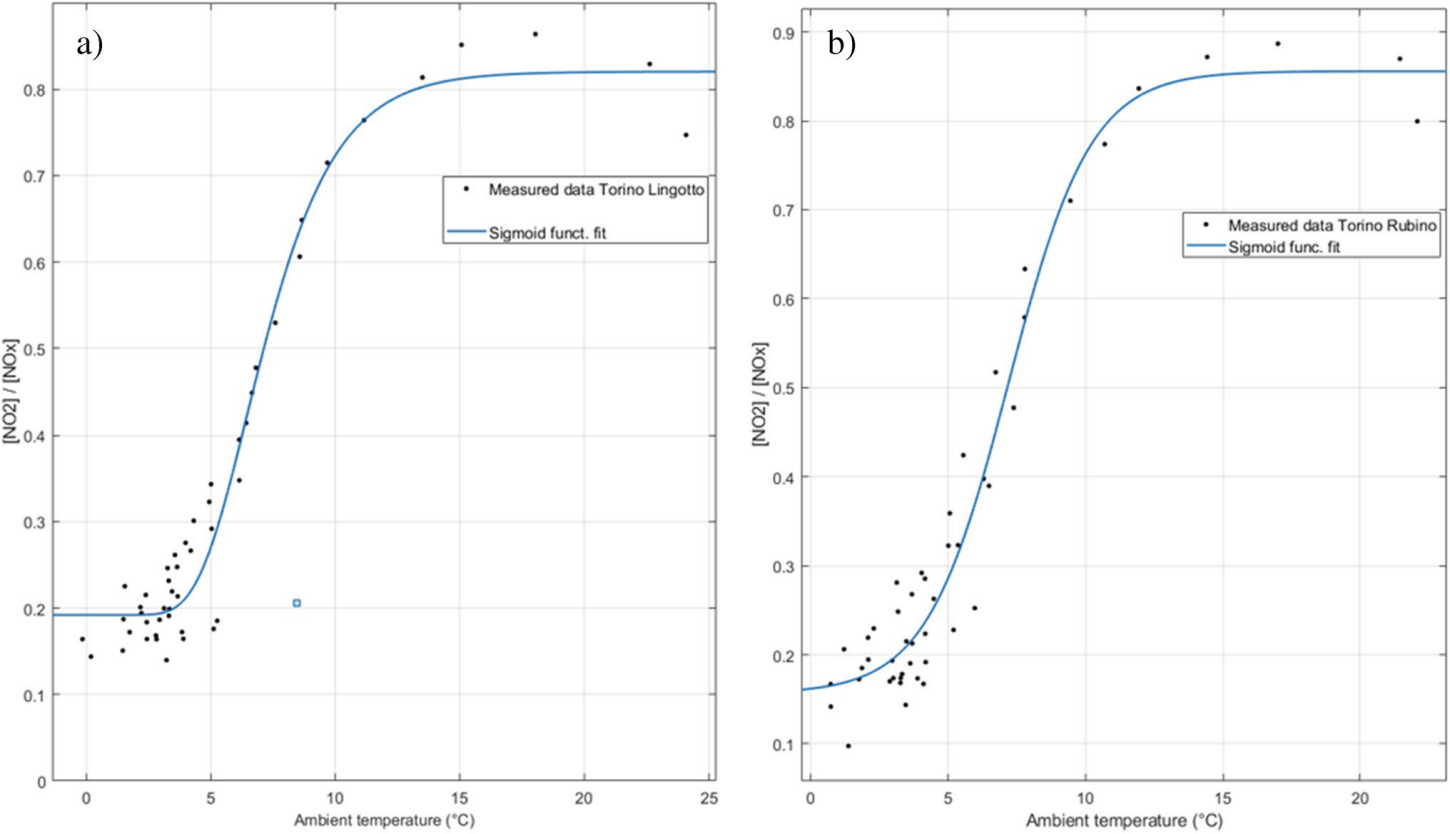
[NO_2_]/[NO_x_] ratio as a function of temperature at **a** Lingotto station and **b** Rubino station in Turin, Italy. The figures report the measures and the data fitting

In this case, y = [NO_2_]/[NO_x_] and x = temperature (°C). The application of a sigmoid function was selected because it better described the physical phenomena of NO_x_ to NO_2_ conversion, which imply the presence of a lower and upper limit. The parameters and stats of this second relationship are reported in Table 4. Both graphs reported in Figure 12 show that for low temperatures, up to 3–5 °C, the [NO_2_]/[NO_x_] ratio is constant or it increases slightly, staying in the range of 0.2–0.3. In the central part (temperatures between 5 and 15 °C), the ratio increases quite rapidly until a peak. In the final part (temperatures above 15 °C), [NO_2_]/[NO_x_] tends to an upper limit corresponding to a ratio of 0.8–0.9. As expected, the complete conversion of NO_x_ to NO_2_ is never reached.

### Comparison of empirical relationships

The empirical relationships obtained in this study were compared with the curves produced by the empirical relationships of previous studies, applied to the concentration values measured in Turin. The averaged values were recalculated and converted from μg/m^3^ to ppb (parts per billion) using a conversion factor of 0.53 for NO_2_.

**Fig. 13:**
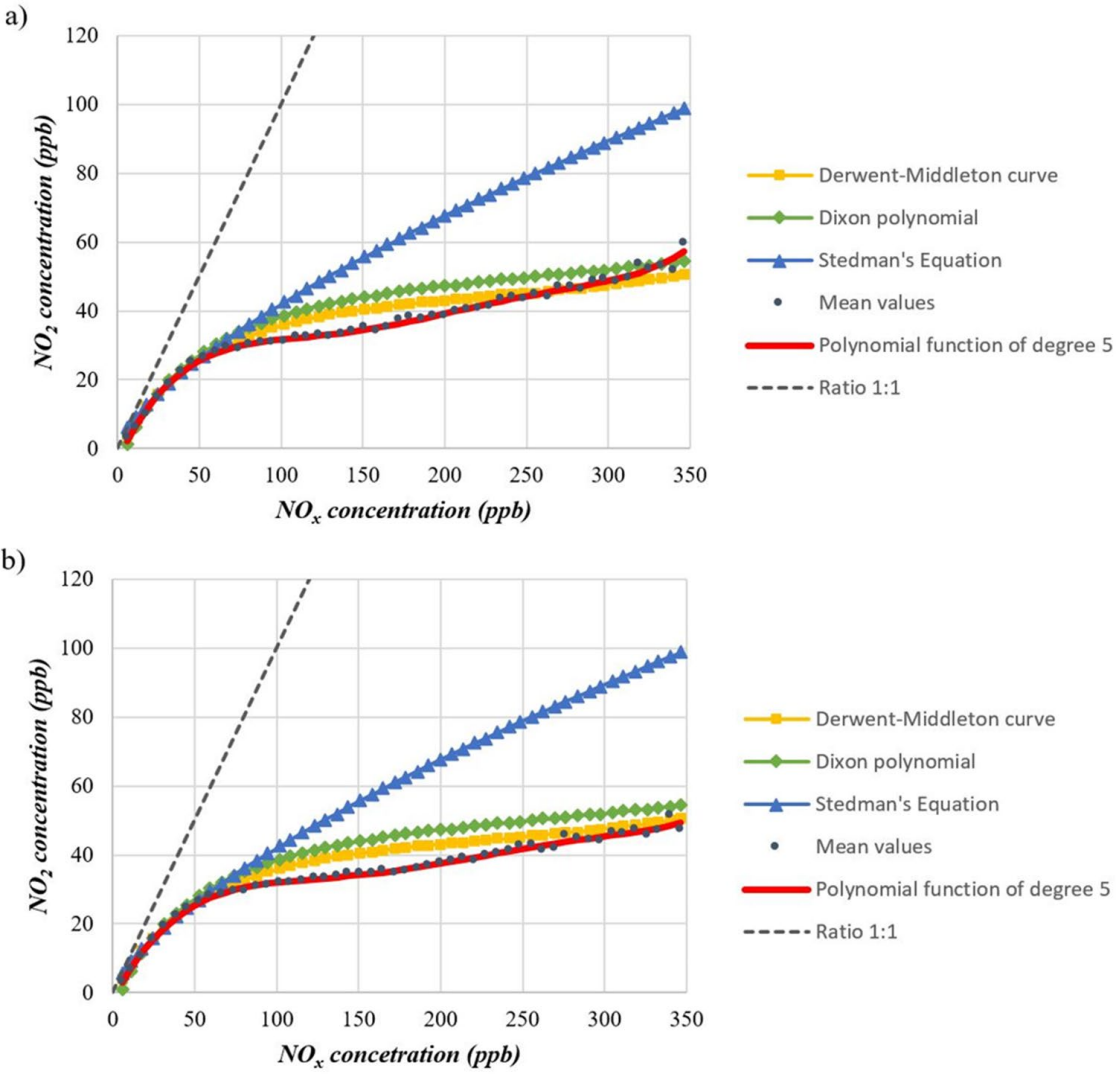
Comparison of overall data curves, **a** Lingotto station and **b** Rubino station

The empirical relations compared were the Derwent-Middleton curve (Eq. 4), the Dixon polynomial (Eq. 7), and the Stedman equation (Eq. 15), with parameter χ chosen arbitrarily equal to 1.76 between the different values given by Stedman. As shown in Figure 13, the curves are perfectly superimposable up to a NO_x_ concentration value of 60 ppb. Above this value, the curves start diverging. The curve of the Stedman equation tends upwards, widening this margin in correspondence with the increase of NO_x_ concentrations. The Derwent-Middleton curve and the Dixon polynomial curve, on the other hand, follow a similar trend, with slightly higher values in favour of the Dixon equation. For both curves, however, there is a rapid increase in NO_2_ concentrations in the range 0 ÷ 50 ppb of NO_x_, followed by a lower increase, which however continues steadily up to the final values observed. The curves related to the 5^th^-degree polynomials obtained in this study are very close to the other curves, revealing a discrepancy of the values in the concentration interval 80 ÷ 230 ppb of NO_x_ for the Lingotto station, and 70 ÷ 280 ppb of NO_x_ for the Rubino station. In both graphs, once the value of 300 ppb of NO_x_ has been exceeded, the curves tend to be closer to the Derwent-Middleton and Dixon curves, with only the Lingotto station showing an upward trend at the end that exceeds the two curves. The values of polynomial coefficients found in this study show a pattern similar to those of previous relationships. Coefficients are higher at degrees 1 and 2, indicating the systematic distance of the concentration of the two species. At higher degrees, the functions show local maxima and minima that fluctuate and tend to a horizontal asymptote around 50–60 ppb NO_2_. This trend confirms the presence of external factors (meteorology and variability of emissions primarily) that perturbate the equilibrium. The accuracy of the models considered in this study is reported in Table 5, which shows the R^2^ and RMSE values for each model against data of Turin city. Since it was specifically elaborated with the datasets of Turin city, the polynomial model shows better fitting than other models for this specific case. However, its extended applicability in other urban areas should be further evaluated. Regarding the application of the polynomial model using ppb units, it must be clarified that such conversion has to be done after the computation of NO_2_ concentration. If users wanted to directly apply Eq. 17 using ppb units, they should change coefficients values in Table 3 accordingly.

## Discussion

This work, analysed the time series of NO_x_, NO_2_, NO, and O_3_ concentrations measured in the period 2015–2019 by two monitoring stations of the Regional Air Quality Monitoring System of the Piedmont Region, both located in the city of Turin, Italy. The study examined the distribution of NO_x_ and NO_2_ concentrations in different time frames, highlighting some differences in the dispersion of the validated data. Specific empirical relationships for the determination of NO_2_ concentration values as a function of NO_x_ concentrations were elaborated. The elaboration of the aforementioned empirical relations was carried out taking inspiration from previous studies carried out by Derwent and Middleton (1996) and by Dixon et al. (2001). Polynomial functions were adapted to the average concentration values returned by the division into classes of 10 μg/m^3^ of NO_x_. All the polynomial functions were derived with a degree 5 approximation for the various time periods examined. The choice of an empirical function to estimate the trend of NO_2_ concentrations is potentially useful for the preliminary data analysis, especially in case of data scarcity. The scatter plots of the two monitoring stations describe a different arrangement of the points, which may be attributable to a different urban context in which the stations are located. The dissonance between a purely residential context (Rubino station) and another characterised by the co-presence of residential buildings and industries of various kinds (Lingotto station), leads to the need to consider a greater contribution to the calculation of the concentrations emitted in an industrial/residential context due to a greater presence of industrial chimneys but also to more intense motorised vehicle transport. This theory is confirmed by several studies (Carslaw and Beevers 2005; Kimbrough et al. 2013; Richmond-Bryant et al. 2017), which highlight the incidence of road transport as one of the main causes of the increase of the [NO_2_]/[NO_x_] concentration ratio in urban areas. This is confirmed by the result obtained, which show a higher [NO_2_]/[NO_x_] ratio at the Lingotto station once the 240 μg/m^3^ NO_x_ value is exceeded. Further differences can be found in the scatter plots relating to the hot and cold seasons, where the distributions are highly dissimilar. The in-depth study, dedicated to NO_x_ and NO_2_ concentrations correlated with the temperature variation, revealed a marked increase of [NO_2_]/[NO_x_] ratio as temperature increases. Higher temperatures are responsible for higher chemical reaction rates (K in Eq. 11), which shift the equilibrium towards higher NO_2_ concentrations. Some considerations should also be expressed on the results obtained from the analysis of the ratio between nitrogen oxides and tropospheric ozone (Figures 4 and 5). It has been observed that, as O_3_ concentration increases, there is a consequent reduction of NO_x_ concentration, due to the chemical reactions of the photo-stationary cycle that takes place between the two species. The observations already reported previously (Cryrys et al., 2012; Jenkin, 2004a, 2004b; Richmond-Bryant, 2017) are confirmed, regarding the composition of the photochemical oxidant OX; i.e., the O_3_ fraction prevails under conditions of low NO_x_ concentrations, and the NO_2_ fraction prevails under conditions of high NO_x_ concentrations. Even though temperature and radiation are the most important parameters regulating photochemical reactions, other meteorological factors, such as wind conditions and air humidity, also contribute to the formation, dispersion and removal of the concurrent species. In the case of Turin, wind speed is generally low and it is not thought to affect the average trend. In other cases, the effect of wind might overcome that of temperature. To account for the whole meteorological conditions, [NO_2_]/[NO_x_] ratios could be analysed as a function of stability conditions in the lower atmosphere (e.g., stability class, mixing height, Monin-Obukhov length). This activity could be the object of future studies.

Regarding the measurement technology, it should be noted that the chemiluminescence method is subject to interferences from a number of sources. The equipment considered in this study is compliant with the specifications set by the European Directive 2008/50/EU (European Union 2008) and its related technical reference standard UNI EN 14211:2012 (UNI EN 2012). Although this equipment was successfully tested for its ability to reject interferences, it is known that some gases (HONO, HONO_2_, PAN, and RONO_2_) can directly alter the amount of light detected by the PMT due to chemiluminescence in the reaction cell (Clemitshaw 2004; Heal et al. 2019). This can either be a gas that undergoes chemiluminescence by reacting with O_3_ in the reaction cell or gas that reacts with other compounds and produces excess NO upstream of the reaction cell. Nevertheless, at present, even though several new technologies are taking place, chemiluminescence is still the reference technique for nitrogen oxide monitoring in ambient air (Sobanski et al. 2021).

The empirical formulations produced in this work on the basis of the observed concentration values proved to be comparable with the empirical relations of previous studies. The evident difference observed in the comparison between the polynomial curves and the Stedman equation can be attributed in the first analysis to a relationship, the latter, which is based on a single parameter that varies according to the urban context to which it refers. The study highlighted the consonance of the empirical polynomial relationships with the concentration data collected, potentially apt for a site-specific application in the analysis of possible future mitigation scenarios. Extending the application of empirical relationships to different urban contexts will be the object of future investigations.

## Conclusion

The planning of efficient actions for the improvement of air quality in urban areas relies on the deep knowledge of the chemical and physical processes connected to pollutant dispersion. Existing air quality monitoring data must be employed in support of such analysis. This study analysed the concentration trends of nitrogen oxides and ozone in the urban area of Turin, Italy, across a 5-year period. The relationships among different species that are involved in the photochemical interactions at the low level of the troposphere confirmed that the reaction equilibrium is affected by several parameters. The production and consumption of NO_x_, NO_2_, and O_3_ may be subject to local changes depending on the source typology and the presence of light and other species. [NO_2_]/[NO_x_] ratio was observed to be strongly affected by temperature. The elaboration and analysis of a set of empirical relationships for the determination of the [NO_2_]/[NO_x_][NO_2_]/[NO_x_] ratio provided preliminary support for the estimation of nitrogen oxides conversion rates. Differences were found between two monitoring stations (Lingotto and Rubino) that are located close one each other. Such a difference was more evident at high NO_x_ concentrations. Thus, the application of these equations for the preliminary estimation of NO_x_ conversion to NO_2_ should be, in principle, restricted to a limited area and a limited range of NO_x_ concentrations. In a second part of the study, the equations obtained by previous studies were fitted to the present dataset. Surprisingly, other equations, except for one, showed similar patterns to the present one, indicating analogies among different urban areas. Starting from the work done in this study, a simplified model for the [NO_2_]/[NO_x_] concentration conversion could be developed, taking into account day/night and seasonal variations, as well as meteorological parameters, temperature and radiation in particular. Nevertheless, as a final remark, it must be pointed out that the adoption of an empirical approach, like that presented in this study, must be considered carefully as it cannot guarantee the same level of consistency of more complex and analytical formulations.

**Table 1:** Correlation and ratio between NO_2_ and NO_x_ ( adapted from Cyrys et al. 2012))

Study area	Correlation between NO_**2**_ and NO_**x**_ (R^2^)	Ratio [NO_2_]/[NO_x_]		
		Regional background	Urban background	Street
Copenhagen	0.94	0.78	0.72	0.58
London / Oxford	0.93	0.39	0.63	0.53
Paris	0.93	0.63	0.64	0.46
Turin	0.94	0.69	0.56	0.52
Rome	0.94	0.59	0.65	0.56
Barcelona	0.93	0.66	0.65	0.57
Athens	0.81	0.62	0.55	0.44

**Table 2:** Validated data for Turin monitoring stations

	Turin-Lingotto	Turin-Rubino
Total validated data	40.235	41.818
Hot season	20.214	20.714
Cold season	20.021	21.080
Daytime	20.027	20.797
Nighttime	20.208	21.021

**Table 3:** Parameters and stats of polynomial fitting (Eq. 17, parameters are unitless)

Parameter	Lingotto station	Rubino station
P1	4.45 10^−11^	2.8 10^−11^
P2	−6.30 10^−8^	−4.5 10^−8^
P3	3.40 10^−5^	2.73 10^−5^
P4	−8.6 10^−3^	−7.8 10^−3^
P5	1.12	1.09
P6	0	0
R2	0.993	0.993
RMSE	1.891	1.712

**Table 4:** Parameters and stats of data fitting of [NO_2_]/[NO_x_] ratio against temperature (Eq. 18, parameters are unitless)

Parameter	Lingotto station	Rubino station
a	0.1471	0.1563
b	0.01215	0.005539
c	0.01774	0.00792
d	0.5223	0.6708
R2	0.9097	0.9604
RMSE	0.06864	0.04734

**Table 5:** Accuracy of the analysed models against Turin datasets

	This study		Derwent-Middleton		Dixon		Stedman	
	Lingotto	Rubino	Lingotto	Rubino	Lingotto	Rubino	Lingotto	Rubino
R^2^	0.993	0.993	0.929	0.963	0.923	0.960	0.940	0.910
RMSE	1.891	1.712	3.019	2.173	3.627	2.606	6.573	7.927

## Data Availability

Data of air quality monitoring station can be found at: http://www.regione.piemonte.it/ambiente/aria/rilev/ariaday/ariaweb-new/
